# Broccoli leaf-derived carbon dots reinforced chitosan/gelatin film as UV-blocking, antioxidant, and antibacterial films for food packaging

**DOI:** 10.1039/d5ra07684f

**Published:** 2025-12-16

**Authors:** Nhung Thi Tran, Thanh Nhan Le, Le Minh Nguyen, Giang Tien Nguyen, Tan Nhiem Ly

**Affiliations:** a Ho Chi Minh City University of Technology and Education 01 Vo Van Ngan Street, Thu Duc Ward Ho Chi Minh City 700000 Vietnam nhungtt@hcmute.edu.vn

## Abstract

Carbon dots (CDs) were synthesized from broccoli leaf waste *via* a hydrothermal process at 180 °C for 8 h and characterized by UV-Vis, PL, FTIR, XRD, TEM, and zeta potential analyses. The CDs exhibited quasi-spherical morphology with diameters of 5–15 nm, a polycrystalline structure composed of amorphous and graphitic domains, and diverse surface functional groups (–OH, –COOH, –NH_2_, C=O, and sulfur-containing moieties), conferring their great aqueous dispersibility and photoluminescent stability under varying pH (2–12) and NaCl concentrations (0.0–2.0 M). The CDs were subsequently incorporated into chitosan/gelatin films at loadings of 0–10% relative to the polymer mass. At 5% loading, the films displayed a great tensile strength of 80.32 MPa, notable 2,2-diphenyl-1-picrylhydrazyl (DPPH) radical scavenging efficacy of ∼90%, and significantly low water solubility (∼13%). Moreover, the composite films exhibited reduced water swelling, enhanced thermal stability, greater UV-blocking capacity, and improved antibacterial activity against *Escherichia coli* and *Staphylococcus aureus* as compared to the neat chitosan/gelatin film. Practical studies demonstrated that the CDs-loaded films effectively protected green apples from UV-induced damage and significantly extended the shelf life of strawberries, outperforming commercial polyethylene (PE) film. All these results highlight the potential of broccoli leaf-derived CDs as effective nanofillers for the fabrication of multifunctional chitosan/gelatin-based food packaging films.

## Abstract 2

Synthesis of broccoli leaf-derived carbon dots and their incorporation into chitosan/gelatin as multifunctional packaging film.

## Graphical abstract

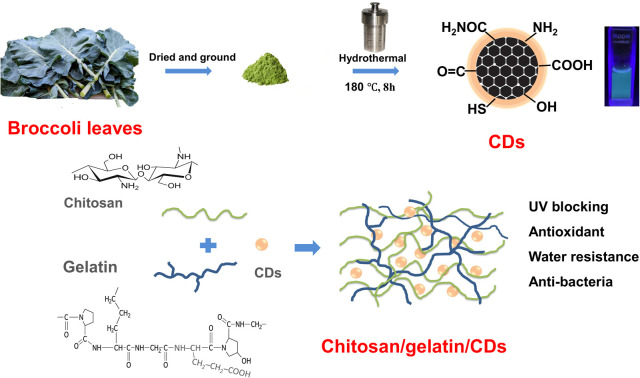

## 1 Introduction

The increasing demand for sustainable and multifunctional active food packaging materials has driven research into biodegradable, non-toxic polymers as promising alternatives to petroleum-based plastics.^1^ Among various biopolymers, chitosan and gelatin are two bio-based polymers which have been intensivelye used as film-forming materials in food and pharmaceutical industries owing to their low cost, abundance, biodegradability, biocompatibility, and intrinsic antioxidant and antimicrobial properties.^2^ However, films prepared solely from chitosan or gelatin often suffer from poor mechanical strength, brittleness, high water sensitivity, and limited functional performance, which restrict their broader application as practical packaging materials.^3^ Blending chitosan and gelatin at appropriate ratios can enhance the mechanical and gas barrier properties of the resulting film through the formation of abundant chemical cross-link networks.^2,4^ In addition, the incorporation of functional nanofillers such as metal and metal oxide nanoparticles, carbon-based materials, essential oils, and plant extracts into chitosan/gelatin films has emerged as an effective strategy to reinforce structural and barrier properties while endowing the film with multiple functionalities.^3,5–7^

Among various nanomaterials employed as nanofillers, heavy metal-free luminescent carbon dots (CDs) have recently gained considerable research attention in numerous applications including sensing, catalysis, optical imaging, phototherapy, and energy storage due to their intriguing optical and electrical properties, low toxicity, rich surface chemistry, and biocompatibility.^8–10^ Unlike inorganic nanoparticles, CDs can be readily synthesized from a wide range of precursors such as small organic molecules, macromolecules, graphite, and biomass resources, *via* diverse techniques.^11,12^ Increasing efforts are now directed toward fabricating CDs from biomass by-products and incorporating them into biodegradable polymers to produce multifunctional, safe, and cost-effective packaging films.^13–16^ Incorporation of CDs has been shown to improve the UV-barrier, antioxidant, antimicrobial, and mechanical properties of such films. For example, Khan *et al.* prepared chitosan/gelatin films with a 1 : 1 weight ratio and incorporated varying amounts of CDs (1 wt%, 2 wt%, and 3 wt%) synthesized from green tea leaves, yielding active films with enhanced tensile strength, antioxidant, antimicrobial, and excellent UV-barrier properties for pork packaging.^2^ A similar study was conducted by Ponnusamy *et al.* recently, in which CDs were synthesized from mango peels and incorporated into a chitosan/gelatin blend at 1 wt%, 3 wt%, and 5 wt% to produce active packaging films for minced pork.^4^ Sul *et al.* also demonstrated that incorporating 3% CDs synthesized from banana peels enhanced the UV-shielding and antioxidant activities of chitosan/gelatin films.^17^

Moreover, heteroatom doping, particularly with N, S, and P, is widely recognized as an effective strategy for modulating the physicochemical properties of CDs.^18^ For example, N-doped CDs exhibit a significant enhancement in fluorescence emission and quantum yield compared with undoped CDs, making them a promising candidate for sensing applications.^19^ Zhang *et al.* also reported improved antioxidant efficacy, increasing from 34.6% for undoped CDs to 71.1% for N-doped CDs and 92.9% for N, S-doped CDs.^20^ This improvement is attributed to the increased polarity and the formation of numerous active sites within the carbon frameworks upon N and S doping. In addition, heteroatom doping also enhances the UV-blocking properties of CDs by modifying their electronic structures and increasing light absorption.^21^ Typically, N-containing compounds (*e.g.*, urea, ammonia, ethylenediamine, tyramine)^19,22^ and S-containing precursors (*e.g.*, sulfuric acid, α-lipoic acid, cysteine)^20,23,24^ are employed to facilitate the incorporation of N and S into CDs.

Herein, we have synthesized N,S-doped CDs using broccoli leaves as a carbon precursor *via* a hydrothermal method at 180 °C for 8 h. Broccoli leaves, which account for ∼70% of the plant biomass, are typically discarded after harvesting the florets, leading to adverse environmental impacts.^25^ Broccoli-derived CDs have been synthesized by several groups and evaluated for their antioxidant and anti-inflammatory activities, as well as for fluorescence-based sensing of hazardous species.^26,27^ Notably, they are rich in amino acids that can serve as natural N and S doping sources for CD synthesis.^25^ Therefore, using broccoli leaf waste as the carbon precursor enables the fabrication of N, S-doped CDs without requiring external dopants.^15^ The obtained CDs were comprehensively characterized by UV-Vis, photoluminescence (PL), X-ray diffraction (XRD), transmission electron microscopy (TEM), and zeta potential analyses. Their colloidal stability and PL emission stability were further examined under different conditions, including varying pH values (2–12), NaCl concentrations (0.0–2.0 M), irradiation times (0–120 min), and storage durations (1–4 weeks). In this study, the CDs were incorporated into a chitosan/gelatin (CG) matrix at a wide loading level ranging from 0 to 10% (relative to polymer mass) for food packaging applications. The effects of varying amounts of CD loadings on the films' optical properties, UV-blocking capacity, mechanical performance, water resistance, thermal stability, antioxidant activity, and antibacterial efficacy were systematically investigated. The antibacterial activity of the CDs-incorporated films against *Escherichia coli* and *Staphylococcus aureus* was evaluated using the filter paper diffusion method. At 5% loading, the composite films exhibited enhanced tensile strength, greater thermal stability, improved water resistance, and great antioxidant performance. Practical assessments also demonstrated that the composite films effectively protected green apples from UV-induced damage and extended the shelf life of strawberries by delaying microbial growth, outperforming commercial polyethylene (PE) cling film.

## 2 Experiments

### 2.1 Chemicals

Gelatin, glycerol (C_3_H_8_O_3_, 99%), acetic acid (CH_3_COOH, 96.4%), sulfuric acid (H_2_SO_4_, 95–98%), methanol (CH_3_OH, 99.7%), hydrochloric acid (HCl, 36–38%), and peptone were purchased from XiLong Scientific Co., Ltd., China. Nutrient Broth was obtained from HiMedia Laboratories Pvt. Ltd., India. 2,2-Diphenyl-1-picrylhydrazyl (DPPH, >97.0%) was supplied by Tokyo Chemical Industry (TCI), Japan. Chitosan (>75% of deacetylation) was purchased from a local manufacturer in Vietnam. All reagents were of analytical grade and used as received without further purification.

### 2.2 Synthesis of carbon dots (CDs) from broccoli leaves

Broccoli leaves were collected from a local farm in Lam Dong province, Vietnam and washed thoroughly with distilled water to eliminate surface contaminants. Subsequently, broccoli leaves were cut into small fragments and dried in an oven at 60 °C until complete dehydration was achieved. The dried leaves were then ground into a fine powder using a mechanical grinder and then stored in an airtight bottle for further use. To synthesize CDs, 3.00 g of the dried broccoli leaf powder was transferred into a hydrothermal reaction vessel containing 60 mL of distilled water. The resulting mixtures were stirred continuously for 30 minutes to ensure homogeneous dispersion. The suspensions were transferred into a sealed autoclave and subjected to hydrothermal treatment in a furnace at 180 °C for 8 hours. After cooling naturally to room temperature, the reaction mixture was coarsely filtered through Whatman no. 101 filter paper to remove solid residues. Subsequently, the supernatant was filtered twice through a 0.22 µm membrane to eliminate large particles. The resulting purified CDs suspension was adjusted to the concentration of 25 mg mL^−1^ and stored in dark, airtight containers at 4 °C for further use.

### 2.3 Fabrication of carbon dots incorporated into chitosan/gelatin film

The film-forming solution was prepared by mixing 5 mL of 8.0 wt% gelatin solution (dissolved in double-distilled water at 35 °C for 15 min), 80 mL of 2.0 wt% chitosan solution (dissolved in acetic acid solution 1% v/v) and an aliquot amount of CDs corresponding to 0.0%, 2.5%, 5.0%, 7.5%, and 10.0% relative to the mass of chitosan and gelatin. The mass ratio of chitosan to gelatin is 4 : 1. Water was added to adjust the total volume of the film-forming dispersion to 115 mL. The dispersion was magnetically stirring for 2 hours, followed by sonication for 30 minutes to remove bubbles. Subsequently, 15 mL of the film-forming dispersion was cast on a Petri dish of 9 cm in diameter and left to completely dried at ambient conditions. After drying, the films were peeled off and stored in a desiccator for 48 hours to achieve moisture stabilization before characterization.

### 2.4 Characterization

#### 2.4.1 Characterization of CDs

The optical properties of the synthesized CDs were collected by UV-Vis spectroscopy (UH3500, Hitachi, Japan) and a fluorescence spectrometer (FluoroMax, JOBIN YVON Horiba, Japan). The particle size was estimated using a transmission electron microscopy (TEM, JEM-2100, Hitachi, Japan), operated at accelerated voltage of 120 kV. Fourier Transform Infrared spectrometer (FT/IR-4700, JASCO, Japan) and energy dispersive X-ray (EDX) spectrometer integrated into scanning electron microscopy (TM4000, Hitachi, Japan) were used to analyze the chemical composition of the CDs.

#### 2.4.2 Film characterization

##### 2.4.2.1 Thickness

The films' thicknesses were recorded by a digital micrometer (SHAHE, 0–25 mm, ± 1 µm, China). The measurements were conducted at 3 random positions and the results were presented as mean ± standard deviation (SD).

##### 2.4.2.2 Optical properties

All investigated films were cut into strips measuring 10 mm × 50 mm. The transmittance spectra were recorded in the range of 190–1000 nm using a UV-Vis spectrophotometer (UH-5300, Hitachi, Japan), with air used as the reference. Film transparency was calculated using the following equation:1
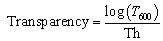
where *T*_600_ (%) is the transmittance at 600 nm, and Th (mm) is the film's thickness. Each measurement was repeated 3 times and the results were presented as mean ± standard deviation.

The UV-blocking capacity of the fabricated chitosan/gelatin/CDs films was calculated based on the following equation:^28^2
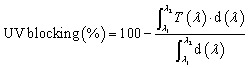
where *T*(*λ*) is the average light transmittance of the film, *λ*_1_ and *λ*_2_ define the wavelength range of interest, for example 200–280 nm for UVC, 280–320 nm for UVB, and 320–400 nm for UVA.

##### 2.4.2.3 Mechanical properties

All investigated films were cut into strips measuring 10 × 50 mm^2^ and mounted in a holder with the gauge length of 24 mm for mechanical testing. Tensile strength (TS, MPa) and elongation at break (EB, %) were measured using a texture analyzer (Brookfield CT3, TA-RCA, Ametek Brookfield, USA) operating at a crosshead speed of 1 mm second^−1^ with a trigger load of 10 mN. Each measurement was repeated five times, and the results were presented as the mean ± standard deviation.

##### 2.4.2.4 Water swelling and water solubility

All investigated films were cut into strips measuring 20 mm × 20 mm and dried in an oven at 45 °C for 24 hours to ensure complete dehydration. The initial dry weights of the films were recorded as *W*_0_. Subsequently, the dried films were immersed in centrifuge tubes, each containing 20 mL of double-distilled water, and incubated at room temperature for 24 hours. After soaking, the swollen films were carefully removed and gently blotted with tissue paper to remove excess surface water. The wet weights were then recorded as *W*_1_. The films were subsequently re-dried in an oven at 45 °C for 24 hours, and their final dry weights were recorded as *W*_2_. All experiments were conducted in triplicate, and the results were reported as mean ± standard deviation.

The water absorption was calculated by the following equation:^29^3
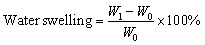


The water solubility was calculated by the following equation:^30^4
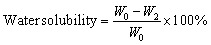


##### 2.4.2.5 Thermal stability

The thermal stability of each investigated film was analyzed using a thermogravimetric analyzer (LINSEIS-STA PT 1600, Germany). The analysis was performed under a nitrogen atmosphere (20 mL min^−1^) with a heating rate of 20 °C min^−1^, over a temperature range of 40 to 700 °C.

### 2.5 Antioxidant activities

The antioxidant activity of the chitosan/gelatin/CDs films was assessed using the DPPH radical scavenging assay. A 0.1 mM DPPH solution was freshly prepared in methanol: H_2_O mixture (2 : 1, v/v). A piece of each investigated film (0.05 g or 0.1 g) was incubated in 4 mL of DPPH 0.1 mM solution contained in dark and airtight vials for 45 minutes. Afterward, the absorbance of the DPPH solutions, with and without film immersion, was measured at 522.5 nm and recorded as *A* and *A*_0_, respectively. The antioxidant activity of the fabricated films was calculated using the following equation:5
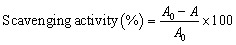


The antioxidant activities of CDs at different concentrations were also determined under the same experimental conditions for comparison.

### 2.6 Antibacterial activities

The antibacterial activity of the chitosan/gelatin/CDs films was examined against *Escherichia coli* (*E. coli*) (ATCC 25913) and *Staphylococcus aureus (S. aureus)* (ATCC 29213) using the disk diffusion method presented by Yang *et al.* with modification.^31^ For the assay, the bacterial strains were first inoculated in nutrient broth medium at 37 °C for 16 h at 150 rpm and serially diluted to a final concentration of 10^8^ cfu mL^−1^ by sterilized saline solution. Afterward, 100 µL of the suspension was evenly spread onto the surface of nutrient agar plates. Sterilized filter paper discs (6 mm in diameter) were immersed in the respective film-forming solutions for 1 h, dried, and placed on the surface of as-prepared agar plates. The plates were then incubated at 37 °C for 24 h, after which the inhibition zones were measured as the diameters of the clear circles formed around the discs.

### 2.7 UV protection of green apples

The UV-blocking performance of the chitosan/gelatin/CDs films was assessed using green apples as a model system under UV light exposure. Freshly harvested green apples of uniform size and color, free from visible defects, were carefully selected, thoroughly washed with water, dried with tissue paper, and placed in paper cups covered with the respective films. A UV lamp (260 nm, 4 W) was positioned 30 cm above the apple surface, and continuous irradiation was carried out for 5 days. Photographs of the apples were taken at 24 h intervals after removing the covering films to monitor their preservation status.

### 2.8 Preservation of strawberries

Freshly harvested strawberries of uniform size, color, and maturity, free from visible defects, were carefully selected. The fruits were thoroughly rinsed with tap water, soaked in saline solution for 5 min, and subsequently washed with double-distilled water. Surface moisture was gently removed using tissue paper, after which the strawberries were individually wrapped with the respective films. Universal glue was used to ensure proper film positioning and airtight sealing. The visual appearance of both unpackaged and packaged strawberries was monitored during storage by capturing photographs at regular intervals. The experiment was conducted in triplicate.

### 2.9 Statistic analysis

All obtained results were statistically analyzed using one-way ANOVA integrated in SPSS software, followed by Tukey's post-hoc test (*p* < 0.05). Different letters in the same experiment indicate statistical difference.

## 3 Results and discussion

### 3.1 Characteristics of broccoli leaf-derived carbon dots

**Fig. 1:**
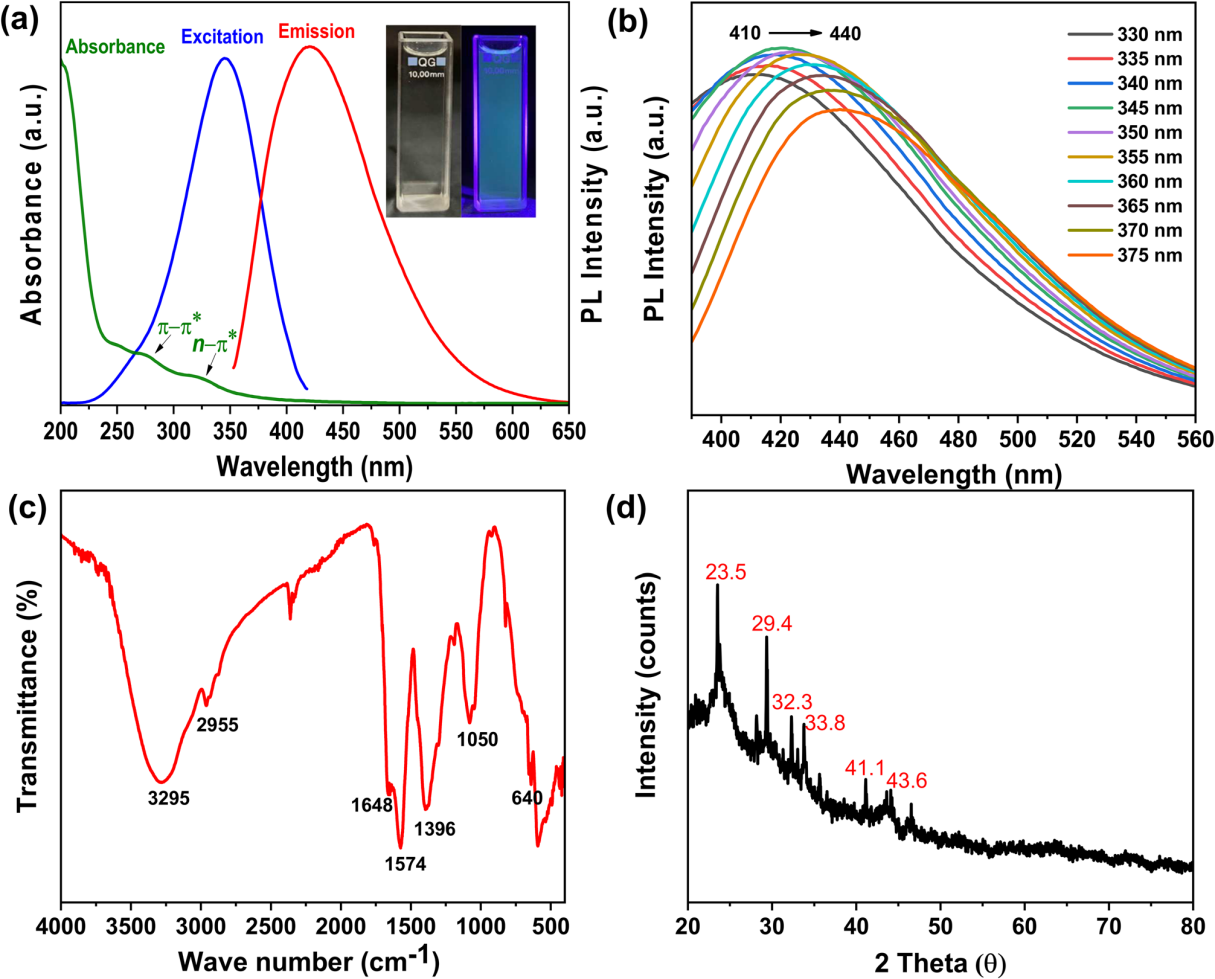
(a) Absorbance, excitation, emission spectra of broccoli leaf-derived CDs. Insets show photographs of the CD solution under ambient light (left) and UV illumination (right), (b) PL emission spectra of the CD solution under different excitation wavelengths, (c) FTIR spectra and (d) XRD pattern of the synthesized CDs.

The CDs were facilely synthesized from broccoli leaves waste powder by hydrothermal method at 180 °C for 8 h. Their characteristics are summarized in Fig. 1. The UV-Vis spectrum (Fig. 1a) displays distinct absorption bands at ∼272 nm and a shoulder at ∼320 nm, which can be ascribed to π–π* transitions of C=C and *n*–π* transitions of C=O/C=N, respectively.^32,33^ The diluted aqueous solution of CDs appears nearly colorless to light yellow under visible light. However, upon excitation at 345 nm, the CDs exhibit a blue-green emission with a maximum at 420 nm. Subsequently, the fluorescence emission properties of the CDs were examined under varying excitation wavelengths (Fig. 1b). As the excitation wavelength increased from 330 to 375 nm, the PL emission maxima gradually red-shifted from 410 nm to 440 nm, indicating excitation-dependent PL behavior. This phenomenon is commonly attributed to the heterogeneity in particle size distribution and the presence of multiple surface defect states associated with various functional groups on the CDs.^34^ In addition, PL stability is a critical factor in evaluating the potential applications of CDs-based materials.^34^ We have investigated the PL stability of the synthesized CDs under different conditions as shown in Fig. S1. In our study, the synthesized CDs exhibited excellent ionic stability, showing no significant change in PL emission intensity over a wide range of NaCl concentrations (0.5–2.0 M). A slight decrease in intensity (∼10%) was observed upon continuous irradiation at 345 nm for 120 min. Nevertheless, the PL spectra remained stable, without notable change in intensity after four weeks of storage. Regarding pH variation, the PL intensity increased markedly from pH 2 to pH 4 and then remained nearly constant between pH 4 to pH 12.

The FTIR spectrum (Fig. 1c) further confirms the presence of multiple functional groups on the surface of the CDs. The strong transmittance band at 3295 cm^−1^ is attributed to the O–H/N–H stretching vibrations, while the band at 2955 cm^−1^ is due to the C–H stretching.^35^ The bands at 1648 cm^−1^, 1574 cm^−1^, 1396 cm^−1^ and 1050 cm^−1^ are assigned to the C=O stretching,^36,37^ N–H bending,^34^ C–N stretching,^36,38^ and C–O–C/C–O stretching vibrations,^37,39^ respectively. In addition, the band at 640 cm^−1^ is characteristic of C–S stretching vibrations.^27^ These results demonstrate the presence of a variety of functional groups, including hydroxyl, carbonyl, carboxylic, amine, and sulfur-containing moieties, on the surface of the CDs. The abundance of hydrophilic groups facilitates the good dispersion of CDs in aqueous media and promotes their interaction with other components during the fabrication of packaging films.

The XRD pattern (Fig. 1d) of the as-prepared CDs exhibits a broad diffraction peak at 2*θ* of ∼23.5° and a negligible hump at 43.5°, which can be assigned to the amorphous carbonaceous materials.^35,40^ Peaks observed at 2*θ* ≈ 29.4°, 32.3°, and 41.2° correspond to graphitic domains with ordered sp^2^ carbon layers.^27,41^ Additional peaks at 33.8° and 35.6° may indicate the formation of a new class of carbon nanomaterials.^27^ These results suggest that the synthesized CDs are polycrystalline, containing both amorphous and graphitic domains embedded within the carbon matrix.

The zeta potential is an important indicator ensuring the dispersion stability of CDs.^42^ As shown in Fig. S2a, the synthesized CDs exhibit a surface potential that is close to neutral at pH 2 (−3.22 mV) and shifts to strongly negative values at higher pH (−50.80 mV at pH 12). The pronounced negative charge values below −20 mV across a broad pH range (pH 4–12) indicate the high colloidal stability of the synthesized CDs.^2^ Consistently, the PL intensity of the CDs remained nearly constant within the same pH range, further confirming their stability. The pH-dependent zeta potential is attributed to the deprotonation of surface functional groups, primarily hydroxyl and carboxylic groups, present on the CDs.^43^

Consistently, the EDX analysis (Fig. S2b) reveals the chemical composition of the CDs which is mainly composed of carbon (50.41 wt%) and oxygen (42.28 wt%), with smaller amounts of nitrogen (6.25 wt%) and sulfur (1.06 wt%). The presence of N and S dopants likely originates from the intrinsic chemical composition of broccoli leaves, a natural biomass precursor. The heteroatom doping, especially N and S, is exploited as a powerful strategy to modulate the optical and electronic properties of CDs.^44^ The additional EDX peaks correspond to Na, Mg, Si, Cl, Ca, and K, which are common trace elements present in plant compositions.

**Fig. 2:**
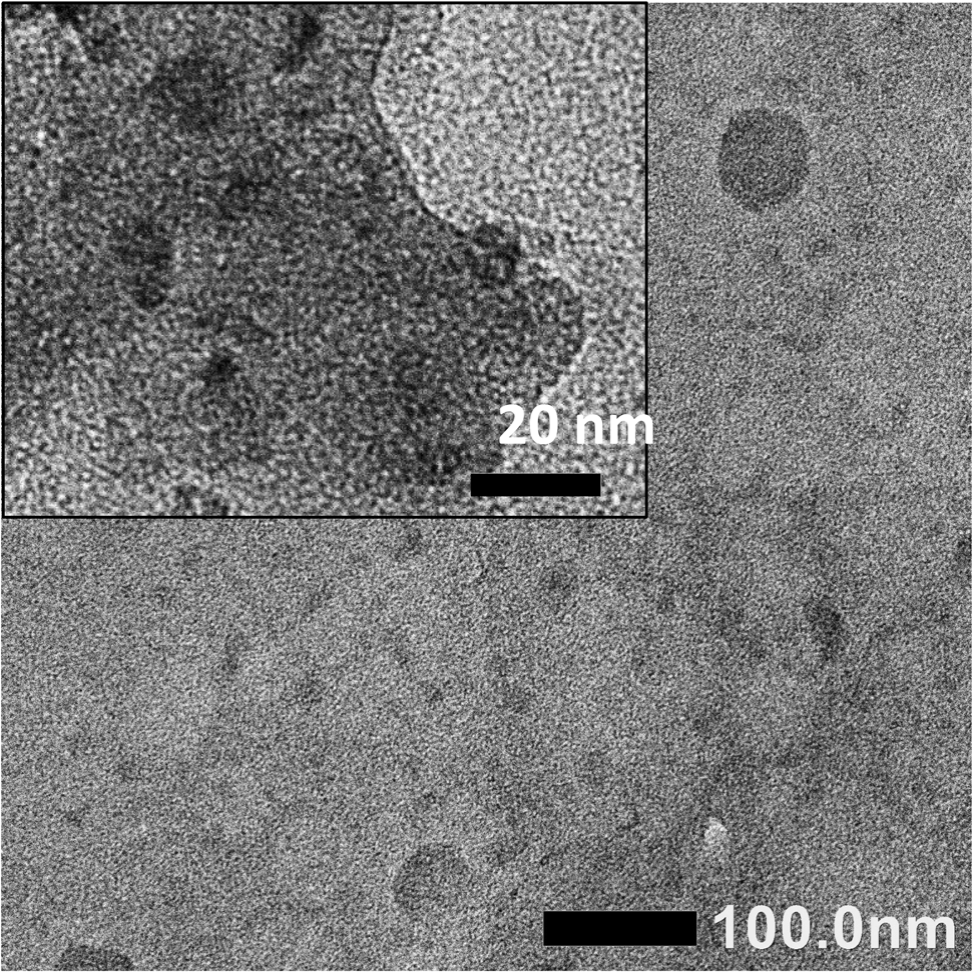
TEM image of the CDs.

The TEM image (Fig. 2) reveals that the as-prepared CDs are spherical with the size ranging at approximately 5–15 nm. In addition, larger particles of approximately 50 nm were also observed, which are attributed to the aggregation of carbon clusters during the growth process.

### 3.2 Characteristics of CDs-loaded CS/GE films

#### 3.2.1 Optical properties

**Fig. 3:**
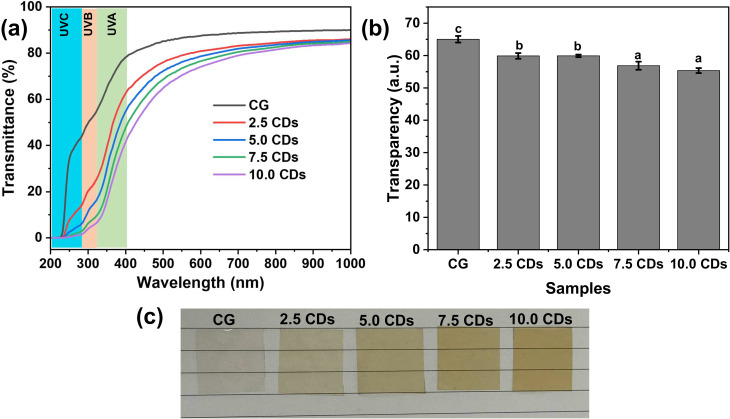
(a) Transmittance spectra, (b) calculated transparency, and (c) photographs of chitosan/gelatin (CG) films incorporating varying amounts of CDs (0–10%).

The transmittance spectra and their corresponding transparency of the chitosan/gelatin films incorporating varying amounts of CDs are displayed in Fig. 3. It is obvious that the transmittance percentage of the neat chitosan/gelatin films decreased progressively with increasing CD content from 2.5 to 10%, particularly in the UV region (200–400 nm). The calculated UV-blocking efficacy of the prepared films is summarized in Table 1. The UV barrier activities significantly increase with increasing the amount of CDs loaded into the chitosan/gelatin film in all regions. In particular, nearly complete blocking (>94%) was observed in the UVC region when the CD loading exceeded 2.5%, and in the UVB region when the CD loading exceeded 7.5%. For the UVA region, the blocking efficiency gradually increased from 33.51% for the pristine CG film to 75.94% at 10% CD loading. The remarkably improved UV-shielding performance of the CD-loaded films compared to the pristine CG film can be attributed to the strong UV absorption of CDs.^14^ Recently, Ponnusamya *et al.* also reported that incorporating 5% mango peel-derived CDs into chitosan/gelatin films remarkably enhanced their UV-blocking capacity, increasing from 56.73% to 98.53% for UVA and from 84.92% to 99.89% for UVB, respectively, which is consistent with our finding.^4^ Furthermore, the transparency of the pristine CG film decreased slightly with the incorporation of CDs (2.5–10%), reaching a maximum reduction of less than 15% at 10.0% CD loading. Nevertheless, the chitosan/gelatin/CDs films retained high optical transparency, as demonstrated by the clear visibility of the underlying photographs. The observed brown color of the film is attributed to the inherent color of the CDs. The excellent film transparency combined with their high UV-blocking performance highlights the potential of chitosan/gelatin/CDs films as promising candidates for applications requiring both UV protection and aesthetic appeal.

**Table 1:** Calculated UV-blocking performance of chitosan/gelatin (CG) film incorporating varying amounts of CDs (0–10%)

Blocking (%)	CG	2.5 CDs	5.0 CDs	7.5 CDs	10.0 CDs
UVA	33.51 ± 0.88^a^	55.81 ± 0.47^b^	62.55 ± 1.85^b^	73.45 ± 2.74^c^	75.94 ± 4.56^c^
UVB	51.54 ± 1.03^a^	80.17 ± 0.83^b^	87.73 ± 1.23^c^	94.36 ± 1.69^d^	95.44 ± 2.32^d^
UVC	81.03 ± 0.62^a^	94.98 ± 0.37^b^	97.83 ± 0.33^c^	99.29 ± 0.32^d^	99.45 ± 0.42^d^

#### 3.2.2 Cross-sectional SEM images

**Fig. 4:**
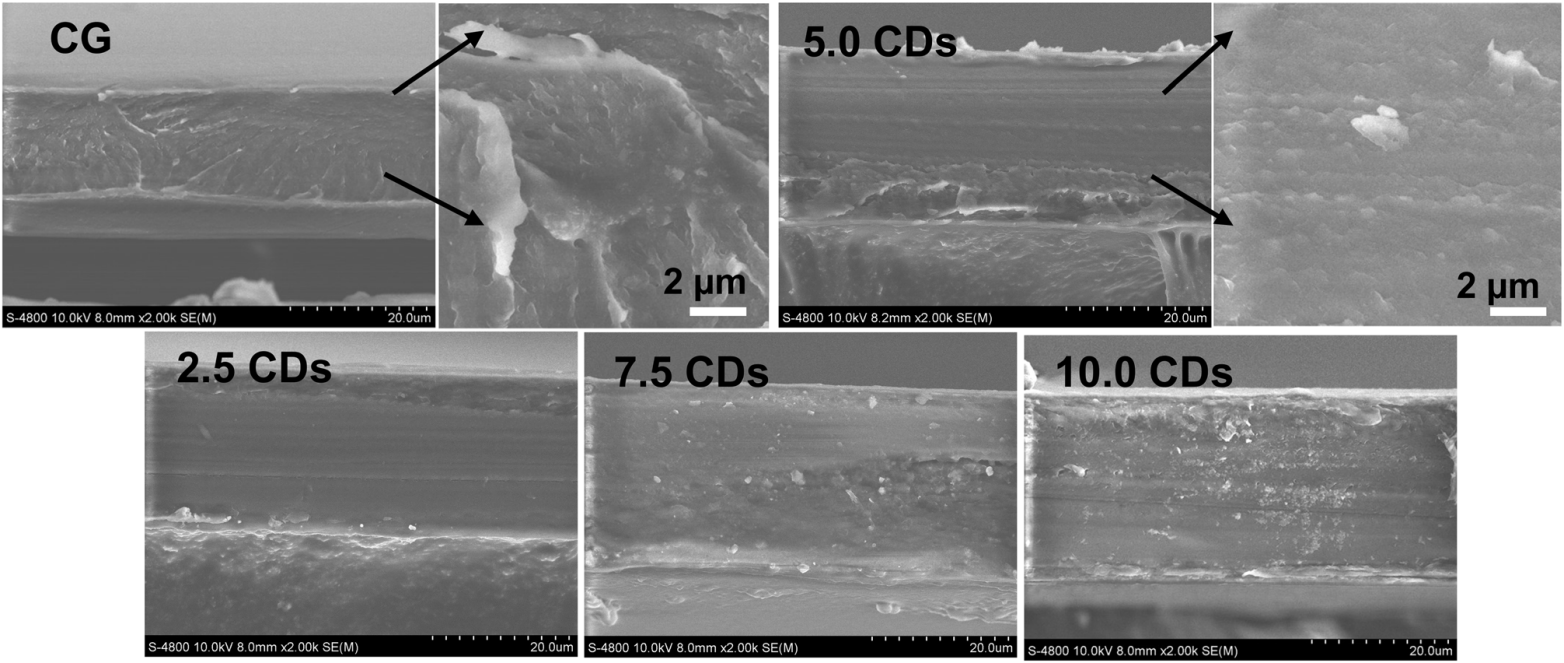
Cross-sectional SEM images of chitosan/gelatin (CG) films incorporating varying amounts of CDs (0–10%).

The SEM cross-sectional images of chitosan/gelatin films incorporating different amounts of CDs are shown in Fig. 4. The pristine chitosan/gelatin film displayed roughened microstructure characterized by small inter-voids and pores. In contrast, the incorporation of 2.5–7.5% CDs resulted in a more compact and coherent structure without visible voids or ruptures as demonstrated by the enlarged image of the film containing 5% CDs. This result suggests good compatibility among chitosan, gelatin and CDs within the film network through the formation of abundant hydrogen bonds. Furthermore, CDs act as nanofillers by occupying voids and pores within the polymer matrix, thereby improving the film's integrity.^4^ However, excessive CD loading at 10% led to the formation of small aggregates and localized voids in certain regions of the film structure.

#### 3.2.3 FTIR spectra

**Fig. 5:**
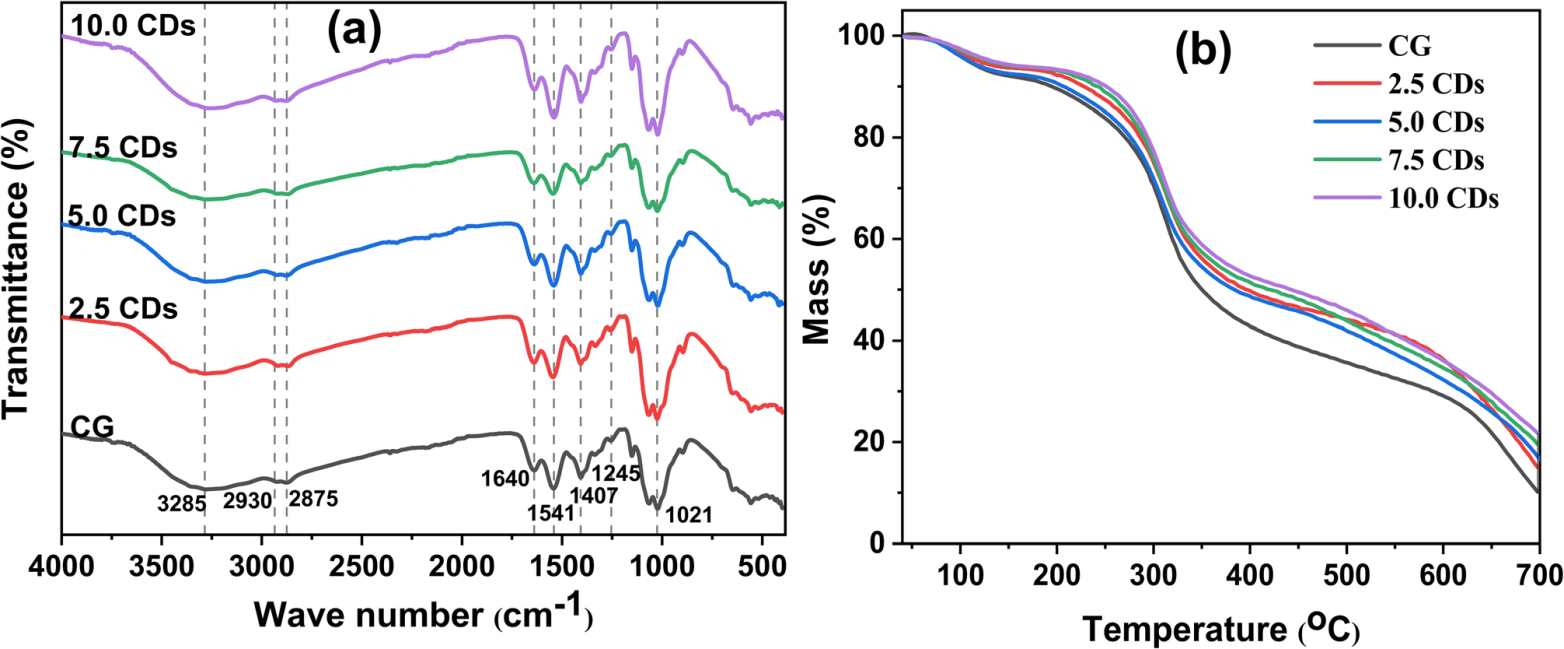
(a) FTIR spectra and (b) TGA surveys and chitosan/gelatin (CG) films incorporating varying amount of CDs (0–10%).

The FTIR spectra of chitosan/gelatin films incorporated with varying CD contents are shown in Fig. 5a. All the films display typical FTIR bands of chitosan and gelatin. Specifically, a broad transmittance band at 3285 cm^−1^ is attributed to the overlapping stretching vibrations of –OH and –NH groups. The bands at 2930 and 2875 cm^−1^ are assigned to the asymmetric and symmetric stretching vibrations of –CH_2_ groups.^45^ Those characteristic bands observed at 1639 cm^−1^, 1541 cm^−1^, and 1245 cm^−1^ are assigned to amide I (C=O stretching coupled with C–N stretching), amide II (out-of-phase combination of N–H in plane bending and C–N stretching), and amide III (in-phase combination of N–H in plane bending and C–N stretching), respectively, which originate from the carbon chains of chitosan and gelatin.^2,46^ The transmittance band at 1407 cm^−1^ is ascribed to the symmetric and asymmetric stretching vibrations of the COO^−^ group.^17^ Furthermore, the band at ∼1021 cm^−1^ is associated with C–O stretching and asymmetric C–O–C stretching vibrations, characteristic of the polysaccharide chains in the chitosan/gelatin polymer matrix.^4^

The CD-incorporated chitosan/gelatin films display a similar pattern with no new band observed compared with the pristine chitosan/gelatin film. This finding indicates the successful incorporation of CDs into the polymer network, primarily through hydrogen bonding between abundant functional groups (–OH, –NH, and C=O) present on both the CDs and the polymer chains.

#### 3.2.4 Thermal stability

Thermal analysis was performed to evaluate the polymer's resistance to thermal degradation and to ensure the material's suitability for thermal-processing methods commonly used in packaging film fabrication (*e.g.*, extrusion, heat sealing).^47,48^ In addition, biomedical materials used in food packaging, UV-blocking systems, drug delivery, wound dressings, or coatings are commonly required to be sterilized at high temperatures (*e.g.* 110–150 °C).^15^ The thermal stability of chitosan/gelatin films with varying CD contents was assessed using thermogravimetric analysis (TGA), as shown in Fig. 5b. All the TG curves display three distinct weight-loss stages. The first stage, occurring at approximately 70–150 °C, is attributed to the evaporation of physically adsorbed water.^2^ The second stage, also the main mass loss, at ∼150–420 °C with a maximum weight-loss rate (*T*_max_) of ∼300 °C is due to the decomposition of polymer chains and incorporated CDs.^17^ The third stage at ∼420–700 °C, characterized by a slow and continuous mass loss, is associated with the carbonization process. The final residue masses at 700 °C of the neat chitosan/gelatin film and the chitosan/gelatin films incorporating 2.5%, 5%, 7.5% and 10% CDs are 9.92%, 14.74%, 17.20%, 19.73%, and 21.84%, respectively. Compared with the neat chitosan/gelatin film, all the CDs-incorporated films exhibit enhanced thermal stability, characterized by lower mass losses during the second and third stages and higher residual mass. A comparable enhancement in thermal stability was also reported by Khan *et al.* for chitosan/gelatin films doped with green tea-derived CDs.^2^ This improvement is attributed to the strong interactions between CDs and the polymer matrix, which strengthen the structural integrity of the films and consequently improve their thermal stability.

#### 3.2.5 Mechanical properties

**Fig. 6:**
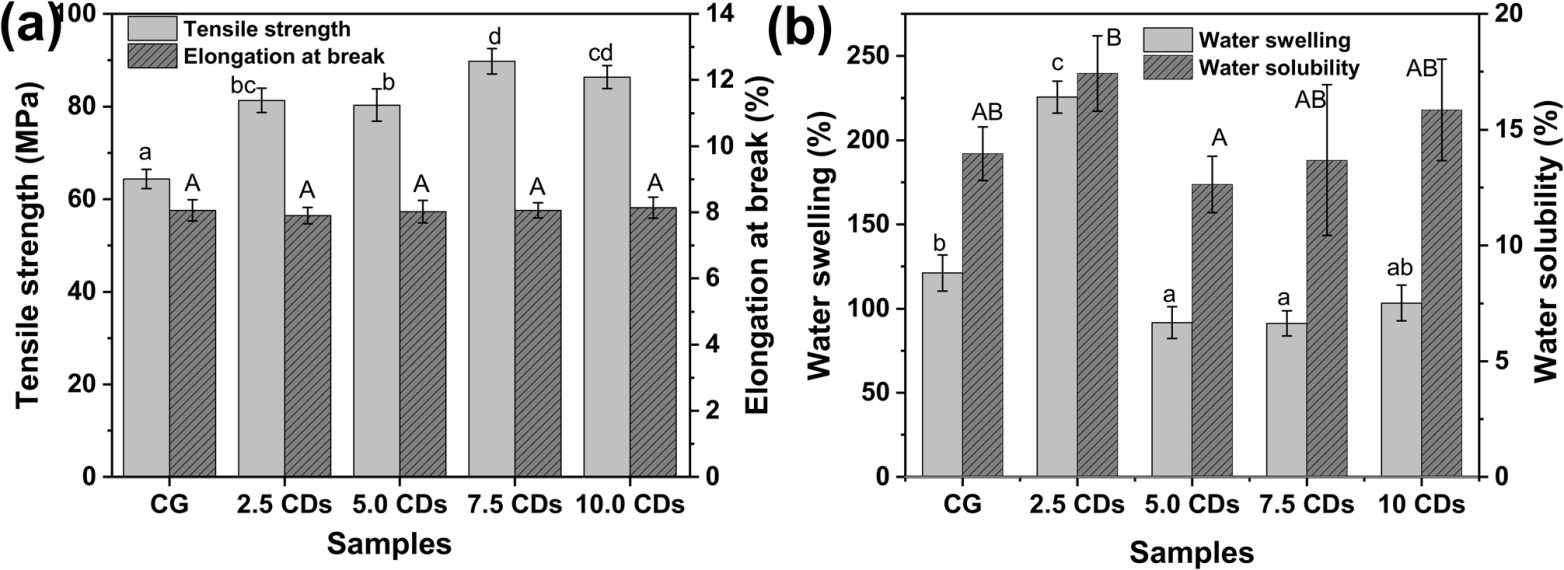
(a) Tensile strength and elongation at break and (b) water swelling and water solubility of chitosan/gelatin (CG) films incorporating varying amounts of CDs (0–10%). Different letters indicate statistical difference (*p* < 0.05).

Mechanical strength is a crucial factor for evaluating the integrity of films for food packaging applications during processing and transportation. The tensile strength (TS) and elongation at break (EB) values of chitosan/gelatin films containing different amounts of CDs are presented in Fig. 6a. In general, TS values increased significantly with increasing CDs content from 2.5% to 7.5% compared with the pristine chitosan/gelatin matrix. The highest TS value, 89.7 MPa, was obtained for the film containing 7.5% CDs, representing an improvement of approximately 40% relative to the pristine chitosan/gelatin film (64.4 MPa). Consistently, Khan *et al.* reported that incorporation of 3 wt% CDs derived from green tea leaves increased the TS of chitosan/gelatin films by 22% (from 65.31 to 79.54 MPa).^2^ A comparable TS value of 81.74 MPa was also recently reported by Ponnusamy *et al.* for the chitosan/gelatin films loaded with 5% mango peel-derived CDs.^4^ This enhancement can be ascribed to the formation of abundant hydrogen bonds and other intermolecular interactions between the functional groups of CDs and the chitosan/gelatin matrix, which promote the development of a denser and more cohesive polymer network.^4^ However, further increasing the CDs content to 10% resulted in a slight reduction in TS value. Excessive addition of nanofillers can disrupt effective polymer–polymer interactions, leading to reduced rigidity of the polymer network and, consequently, diminished TS of the films.^49^

In contrast, the EB values showed no significant differences among the samples, remaining consistently low at ∼8%, which is comparable to the values reported by Tagrida *et al.* for chitosan/gelatin films prepared with different chitosan/gelatin blending ratios.^50^ The strong intermolecular interactions between chitosan and gelatin restrict the mobility and flexibility of polymer chains, thereby accounting for their low EB values observed.

#### 3.2.6 Water swelling and water solubility

The water swelling and water solubility behaviors of chitosan/gelatin films incorporated with varying amounts of CDs are shown in Fig. 6b. The pristine chitosan/gelatin film exhibits water swelling ∼120%. Upon the incorporation of 2.5% CDs, the water swelling increased sharply, reaching the highest value of ∼230%. This pronounced increase can be attributed to the high density of hydrophilic functional groups (*e.g.*, hydroxyl, carboxyl, and amino groups) on the surface of CDs, which enhance water affinity and facilitate the penetration of water molecules into the polymer matrix. However, further increasing the CD content to 5.0–7.5% resulted in a substantial reduction in water swelling (∼90%), followed by a slight increase at 10% (∼104%). This reduction suggests that at higher concentrations, CDs strengthen intermolecular interactions within the chitosan/gelatin matrix and act as nanofillers by occupying internal voids and pores, thereby restricting water uptake. The slight increase observed at 10% may be attributed to the aggregation of CDs at high concentrations, which disrupts the homogeneity of the polymer network and generates localized defects that promote water penetration.

In contrast, the water solubility of the chitosan/gelatin films remained largely unaffected by the addition of CDs, with the lowest value (∼13%) observed at 5% CD loading. For comparison, Tagrida *et al.* reported a much higher water solubility (19.53–74.41%) in chitosan/gelatin films incorporated with betel leaf ethanolic extract at various concentrations.^50^ This difference may be ascribed to the absence of adding glycerol as a plasticizer in the present film formulation. Moreover, compared with other biodegradable polymer films, the chitosan/gelatin-based films in this study exhibited markedly lower water solubility. For example, Chaves *et al.* reported a water solubility of approximately 75% for a pectin/starch/PVA film,^51^ whereas Zhang *et al.* observed water solubility of nearly 100% for pristine sodium alginate films.^52^

The hydrophilicity of the film surfaces was also evaluated by measuring their contact angles, Fig. S3. Although the contact angle slightly increased when the CD content reached 5% and then decreased at 10%, no significant differences were observed across the CG films incorporating varying amounts of CDs. At moderate CD content, interactions between the CDs and the polymer slightly reduce the number of hydrophilic groups on the film surface, which would tend to increase the contact angle; however, higher CD content introduces abundant hydrophilic groups, resulting in an overall contact angle that remains largely unchanged.^4,15^

In addition, water vapor transmission rate (WVTR) is an important factor for evaluating the preservation efficacy of packaging films.^53^ The WVTR values of chitosan/gelatin films incorporating varying amounts of CDs are presented in Fig. S4. Our results indicate that the chitosan/gelatin/CDs films exhibited approximately a 10% reduction in WVTR compared with the neat chitosan/gelatin film. This improvement can be attributed to the nanofiller effect of the incorporated CDs, which hinders the diffusion of water vapor through the film. Overall, all these results demonstrate the enhanced water resistance of the fabricated chitosan/gelatin-based films.

### 3.3 Antioxidant activities

**Fig. 7:**
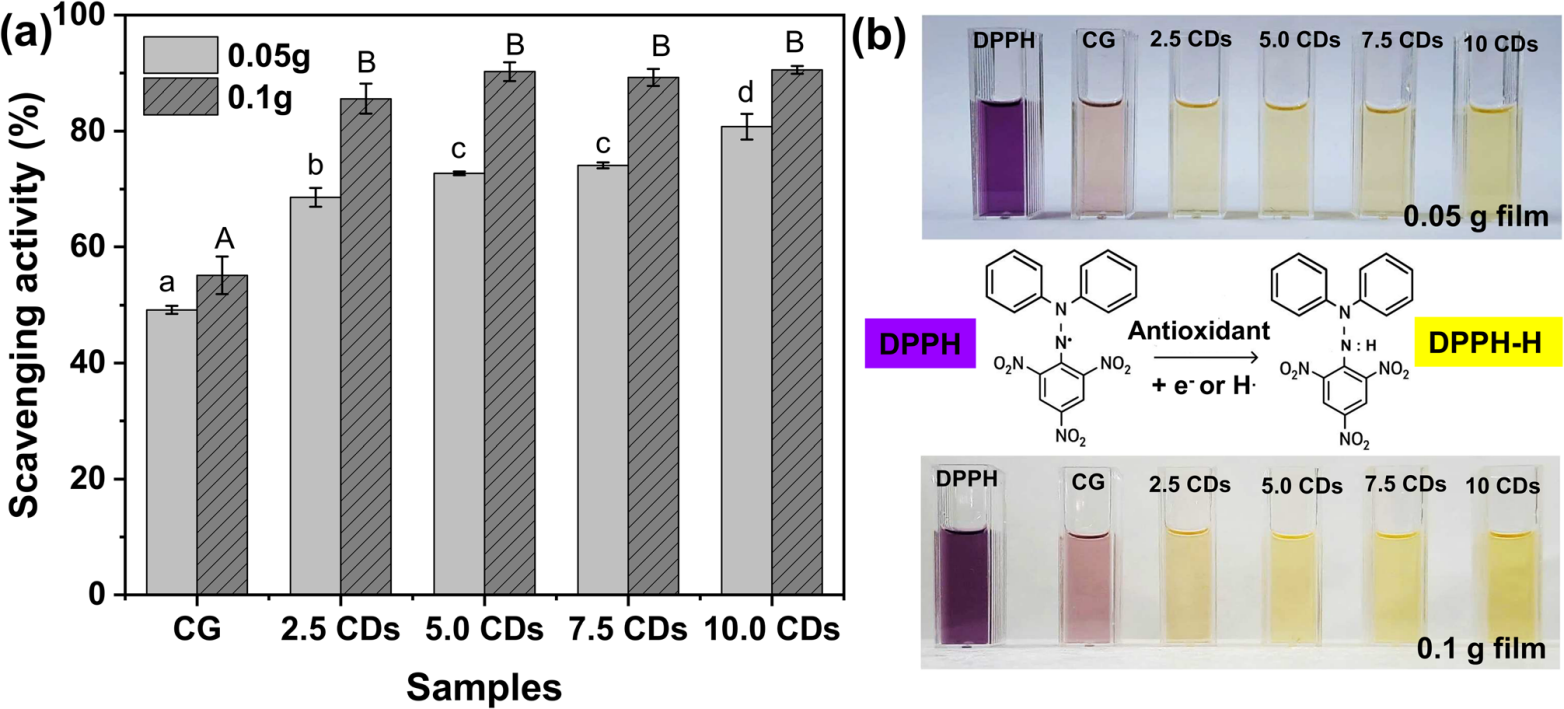
(a) DPPH scavenging efficacies of 0.05 g and 0.1 g chitosan/gelatin (CG) films incorporating varying amounts of CDs (0–10%), and (b) photographs of DPPH solutions exposed to 0.05 g and 0.1 g films along with a schematic depiction of the DPPH scavenging mechanism. Different letters indicate statistical difference (*p* < 0.05).

The antioxidant activities of the CDs-incorporated chitosan/gelatin films were evaluated using the DPPH free radical scavenging assay, as shown in Fig. 7. DPPH is a stable free radical characterized by a deep purple color in its oxidized form, with a typical absorbance maximum at 522.5 nm. It is widely employed as a model system to assess the radical-scavenging ability of antioxidants. In the presence of electron- or hydrogen-donating agents, DPPH is reduced to its hydrazine derivative (DPPH-H), resulting in a color change from purple to yellow.^54^

The pristine chitosan/gelatin film exhibited scavenging capacities of ∼50% and ∼55% when 0.05 g and 0.1 g of the film were added to the DPPH solution, respectively. The inherent antioxidant activities of chitosan and gelatin are attributed to the presence of electron and proton-donating functional groups in their chemical structures, which is in consistent with previous reports.^2^ The incorporation of CDs further enhanced the antioxidant performance of the composite films. Specifically, when adding 0.05 g of film, the scavenging efficacy increased gradually, reaching maxima at ∼80% as the CDs content increased from 2.5% to 10%. This improvement is ascribed to the abundant electron- and proton-donating functional groups in CDs, which synergistically contributed to the radical scavenging effect.^55,56^ When the film mass increased to 0.1 g, the antioxidant activity of CDs-loaded films increased sharply to ∼85% at 2.5% CDs loading and then plateaued at ∼90% with further increases in CD loading up to 10%. This finding indicates that a moderate CDs content is sufficient to achieve saturated antioxidant activity.

For comparison, the antioxidant capacity of the pristine CDs at various concentrations and incubation times was also investigated, Fig. S5. The scavenging efficiency of CDs increased with both concentration and incubation time. A rapid increase was observed within 5 minutes of incubation, followed by a gradual rise until reaching a plateau after 30 minutes. The antioxidant activity also improved significantly as the concentration increased from 100 to 400 µg mL^−1^, whereas further addition up to 500 µg mL^−1^ did not enhance the activity significantly. The maximal scavenging efficiency of the CDs solution was ∼42% obtained at 500 µg mL^−1^ which is relatively lower compared to other studies.^57,58^ For example, Shaik *et al.* reported that N/S-doped CDs synthesized from tartaric acid and thiourea exhibited scavenging activities ranging from 18.2% to 93.2% as the concentration increased from 0.02 to 0.103 mg mL^−1^, with a plateau reached beyond 0.075 mg mL^−1^.^59^

By combining the intrinsic antioxidant activity of the chitosan/gelatin polymeric matrix (∼50–55%) with the contribution from CDs, the chitosan/gelatin/CDs films achieved a maximal scavenging activity of ∼90%. Similarly, Kurek *et al.* also reported the DPPH scavenging efficacy of 88% for the chitosan/gelatin film enriched with gallic acid and orange essential oils.^60^ A lower DPPH scavenging efficacy of 63.87% was reported by Khan *et al.* for the chitosan/gelatin film incorporated with 3% green tea-derived CDs.^2^ The remarkable antioxidant performance of the chitosan/gelatin/CDs films in this study highlights their potential as highly effective antioxidant materials.

### 3.4 Antibacterial activities

**Fig. 8:**
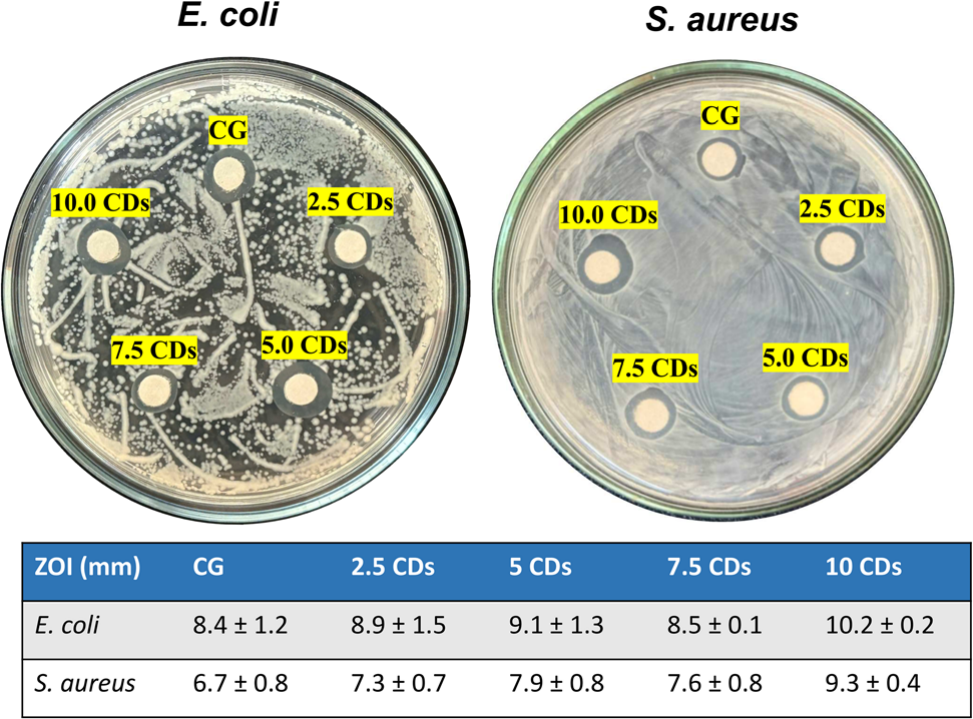
Antibacterial activity of chitosan/gelatin (CG) films incorporating varying amounts of CDs (0–10%) against *E. coli* and *S. aureus*, with corresponding measured zone of inhibition (ZOI) diameters. Note: the ZOIs include the paper disc's diameter (6 mm).

The antibacterial activity of the chitosan/gelatin-based films against *E. coli* and *S. aureus* was evaluated using the disk diffusion method, Fig. 8. Clear zones of inhibition (ZOI) with diameters of 8.4 mm and 6.7 mm were observed for *E. coli* and *S. aureus*, respectively, which can be attributed to the intrinsic bactericidal properties of chitosan.^3^ In general, the antibacterial activities increased with the CD loading for both bacterial strains although the differences are not significant. Specifically, the ZOI increased from 8.4 mm to 10.2 mm for *E. coli* and from 6.7 mm to 9.3 mm for *S. aureus*, respectively, as the loaded amount of CDs rose from 0.0% to 10.0%. Zhao *et al.* also reported ZOIs of 9.89 and 10.02 mm against *E. coli* and *S. aureus*, respectively, for the PVA film incorporating CDs synthesized from banana paste.^14^ Interestingly, a slight decrease in ZOI was observed at 7.5% CD loading for both bacterial strains. It can be attributed to the strong interactions between CDs and the polymeric matrix at this loading level. This observation is consistent with the TS results, where the film containing 7.5% CDs exhibited the highest TS value, reflecting reinforced structural integrity.

The antibacterial activities of carbon dots (CDs) are strongly influenced by their structure, surface functionalization, and applied dosage.^61^ For example, Zhang *et al.* reported that N-doping imparts CDs with a highly positive surface potential, which facilitates strong electrostatic interactions with bacterial cells and thereby enhances their bactericidal efficiency.^62^ Liu *et al.* demonstrated that CDs synthesized from dragon fruit exhibited an inhibition rate of approximately 90% against both *E. coli* and *S. aureus* when the CD concentration reached 8 mg mL^−1^.^16^ In contrast, Prathap *et al.* found that CDs derived from *Prosopis juliflora* leaves showed no visible inhibition zone (ZOI) against *E. coli*, while exhibiting a ZOI of 18 mm and a minimum inhibitory concentration (MIC) of 1.5 mg mL^−1^ against *S. aureus*.^63^ Conversely, Wei *et al.* reported that CDs synthesized from the natural *Gynostemma* plant produced no detectable ZOI against either *E. coli* or *S. aureus*.^64^ Although the antibacterial mechanism of CDs has not been fully elucidated, studies suggest that their ultra-small size and abundant surface functional groups allow them to interact closely with bacterial cells, penetrate the cell membrane, and disrupt metabolic processes, primarily through the generation of reactive oxygen species (ROS), which ultimately leads to cell death.^4,17^ The limited antibacterial activity of the chitosan/gelatin/CDs films may be ascribed to the low CD loading in the polymer matrix and their strong interactions, which hinder effective contact between CDs and bacteria.

### 3.5 Preservation of green apples under UV exposure

**Fig. 9:**
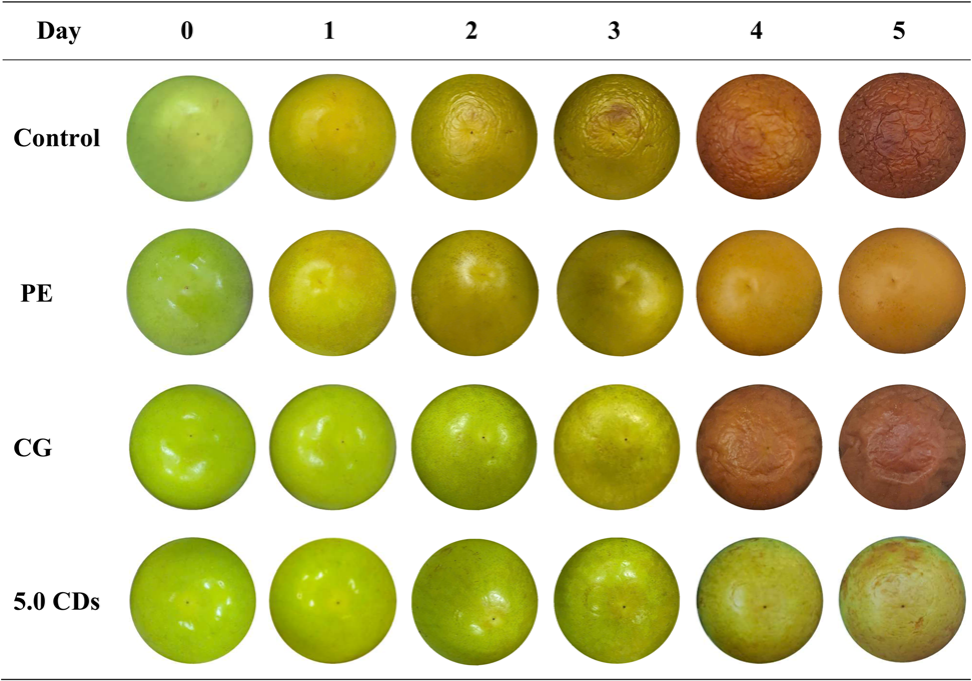
Photographs of green apples over time when unpacked and when packed with polyethylene (PE) cling film, chitosan/gelatin (CG) film, and CG film loaded with 5.0% CDs under UV light illumination.

The photographs of green apples, both unpackaged and packaged with various film types, are presented in Fig. 9. From observation, it is obvious that the apples packaged with the CDs-incorporated film maintained better freshness and colors with minimal signs of UV-induced damage compared with the unpackaged fruits and those packaged with either commercial polyethylene (PE) cling film or pristine chitosan/gelatin films.

### 3.6 Preservation of strawberries

**Fig. 10:**
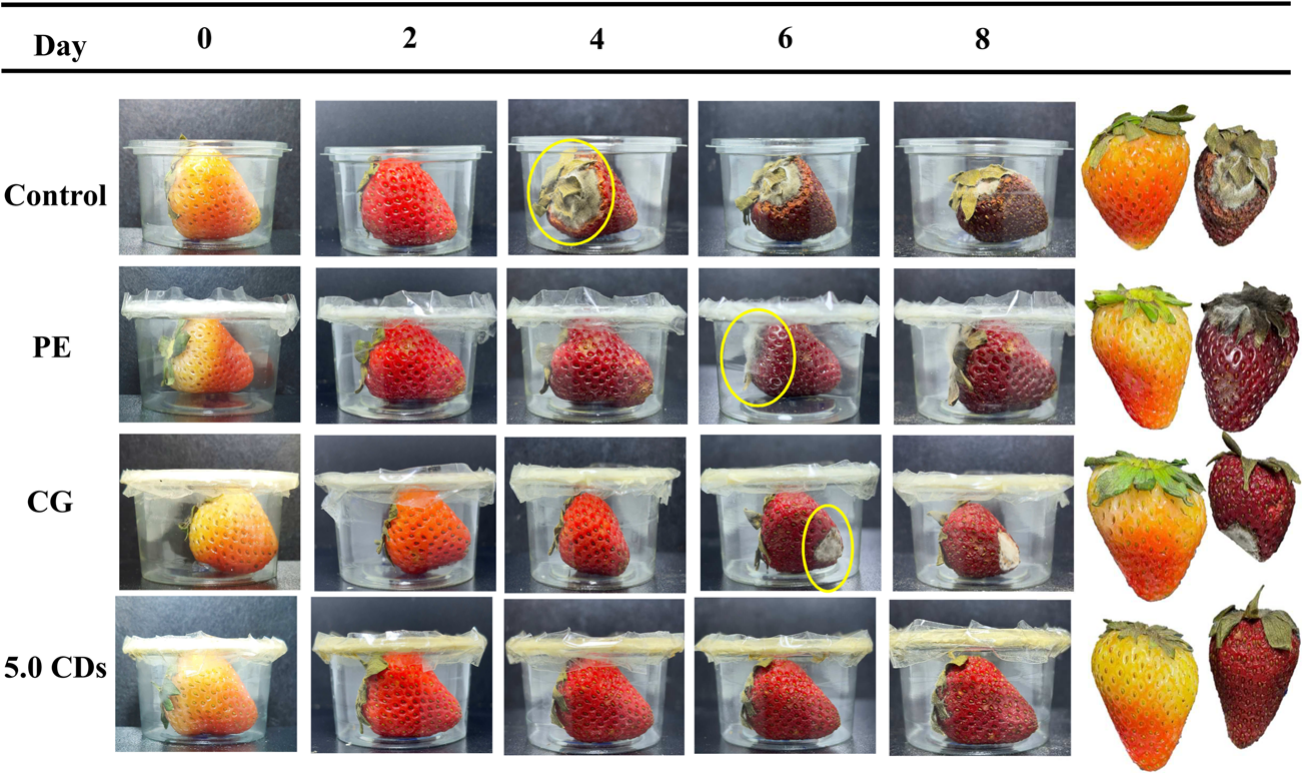
Photographs of strawberries during storage time when unpacked and when packed with polyethylene (PE) cling film, chitosan/gelatin (CG) film, and CG film loaded with 5.0% CDs.

The photographs of strawberries packed with different film types are presented in Fig. 10. Strawberries are highly nutritious fruits but are perishable, primarily due to weight loss, rapid respiration rate, and most importantly, microbial contamination.^65^ Microbial growth became visible on day 4 in unpackaged fruits, whereas it was delayed until day 6 in strawberries packaged with either commercial polyethylene (PE) cling film or the neat chitosan/gelatin film. Notably, strawberries packaged with the chitosan/gelatin film containing 5% CDs showed no visible microbial growth, even after 8 days of storage. Moreover, the fruits packaged with the chitosan/gelatin/CDs film exhibited greater freshness and color retention compared with both the unpackaged fruits and those packaged with PE or chitosan/gelatin films. The enhanced preservation effect of the chitosan/gelatin/CDs films can be attributed to their ability to act as effective barriers to gases, moisture, and bacteria, thereby slowing respiration, reducing water loss, and preventing bacterial invasion. These findings clearly demonstrate the significant potential of CDs-incorporated chitosan/gelatin films for extending the shelf life of fresh fruits.

## 4 Conclusion

In summary, the CDs were successfully synthesized from broccoli leaf *via* a hydrothermal method at 180 °C for 8 h. The CDs exhibit a quasi-spherical morphology with an average size of 5–15 nm and a polycrystalline structure. The surface of CDs was enriched with diverse functional groups, including hydroxyl, carbonyl, carboxyl, amine, and sulfur-containing moieties, which contribute to their excellent aqueous dispersibility and high photoluminescent stability under varying pH and NaCl concentrations. The CDs were subsequently incorporated into a chitosan/gelatin matrix at loadings of 0–10%. The resulting composite films exhibited enhanced mechanical properties, improved thermal stability, reduced water swelling and water solubility, and enhanced antioxidant activity compared with the pristine chitosan/gelatin film. The incorporation of CDs also improved the antibacterial activity of the films against *E. coli* and *S. aureus*, although the effect was not pronounced. Practical evaluations further demonstrated that the CDs-incorporated films provided improved UV-blocking capacity, effectively protecting green apples from UV-induced damage. In addition, the films successfully extended the shelf life of strawberries, outperforming commercial PE cling film. However, this study lacks an investigation of the antibacterial activity of the CDs alone and the films' oxygen permeability or other gas-barrier properties, all of which are essential for providing a more comprehensive understanding of the films' performance in food-packaging applications. Overall, this study demonstrates a promising strategy for utilizing biomass sources rich in amino acids, proteins, or sulfur-containing compounds as effective precursors for synthesizing heteroatom-doped CDs with enhanced optical properties. In addition, the findings highlight the strong potential of incorporating biomass-derived CDs into chitosan/gelatin films to develop multifunctional, sustainable, and cost-effective packaging materials, providing a viable pathway toward sustainable food preservation and green packaging technologies.

## Author contributions

Nhung Thi Tran: conceptualization, methodology, data acquisition and analysis, resources, manuscript preparation. Thanh Nhan Le: methodology, data acquisition and analysis, figure organization, manuscript preparation. Le Minh Nguyen: methodology, data acquisition and analysis, figure organization, manuscript preparation. Giang Tien Nguyen: figure organization, manuscript preparation. Tan Nhiem Ly: figure organization, manuscript preparation.

## Conflicts of interest

The authors declare no conflicts of interest.

## Supplementary Material

RA-015-D5RA07684F-s001

## Data Availability

The data supporting this manuscript have been included as part of the supplementary information (SI). Supplementary information: Fig. S1–S5 and futher experimental details. See DOI: https://doi.org/10.1039/d5ra07684f.
