# Supplementary material for: Broccoli leaf-derived carbon dots reinforced chitosan/gelatin film as UV-blocking, antioxidant, and antibacterial films for food packaging
Source: RSC Adv. 2025 Dec 16;15(58):50089–102. doi: 10.1039/d5ra07684f (PMC12706845; doi:10.1039/d5ra07684f)
Supplement: RA-015-D5RA07684F-s001 [file RA-015-D5RA07684F-s001.pdf]

Supplementary Information (SI) for RSC Advances.
This journal is © The Royal Society of Chemistry 2025

Supporting information

Broccoli Leaf-Derived Carbon Dots Reinforced Chitosan/ Gelatin Film as UV-Blocking, Antioxidant, and Antibacterial Films for Food Packaging

Nhung Thi Tran[1*] Thanh Nhan Le[1], Le Minh Nguyen[1], Giang Tien Nguyen[1], Tan Nhiem Ly[1]

[1]Ho Chi Minh City University of Technology and Education, 01 Vo Van Ngan Street, Thu Duc Ward, Ho Chi Minh City 700000, Vietnam

Corresponding author: Nhung Thi Tran, email: nhungtt@hcmute.edu.vn

Evaluating the PL emission stability of the synthesized CDs

To evaluate the PL emission stability of the synthesized carbon dots (CDs), we have conducted a series of experiments under different environmental conditions:

For ionic stability, aqueous dispersions of CDs (0.02 mg/mL) were prepared in NaCl solutions at the concentrations of 0.0 M, 0.5 M, 1.0 M, 1.5 M, and 2.0 M, and their photoluminescence (PL) spectra were recorded at an excitation wavelength of 345 nm.

To examine pH stability, the pH of the CDs dispersions (0.02 mg/mL) were adjusted pH 2, pH 4, pH 6, pH 8, pH 10, and pH 12 using NaOH solution (1.0 M) and HCl solution (1.0M). The PL spectra were collected with the excitation wavelength at 345 nm.

The stability of the CDs solution (0.02 mg/mL) was also assessed by recording their PL spectra during 1 month of storage (4 °C, dark and airtight bottles).

The photo-stability under UV irradiation was evaluated by continuously exposing the CDs dispersions (0.02 mg/mL) to a UV LED lamp (λ = 345 nm, 6 W) for 120 min, during which PL spectra were measured at 30 min, 60 min, 90 min, and 120 min.

Measuring the contact angles of the films:

The contact angle of the chitosan/gelatin-based films was measured using a modified protocol derived from previously published methods [1, 2]. Briefly, each film sample was cut into a strip measuring 2 cm × 5 cm and mounted onto a glass slide. A micro-syringe was used to dispense 5 μL glycerol droplets at five discrete positions on the film surface. Due to the water-absorbing nature of biopolymer-based films, glycerol was selected as the probing liquid to minimize droplet

distortion and provide more stable, reliable profiles. The glycerol droplet images were captured using a digital camera, and their profiles were subsequently analyzed with ImageJ software to determine the corresponding contact angles. All data were statistically evaluated and are reported as mean ± SD. It should be noted that the results presented here provide a relative comparison among the film types rather than absolute quantitative values.

Measuring water vapor transmission rate (WVTR)

The water vapor transmission rate (WVTR) of the prepared films was evaluated according to a previously described procedure [3] with modifications. In brief, 10 mL of double-distilled water was introduced into glass vials with an orifice diameter of approximately 2 cm. Each vial opening was completely sealed with the investigated film and secured with Teflon tape to ensure proper placement and airtight sealing. The initial weight of each sealed vial was recorded as W_0. The vials were then incubated in an oven at 45 °C for 20 hours, after which the final weight (W_1) was measured. The higher temperature of 45 °C was used to accelerate the water evaporation process. The WVTR was calculated based on the following equation: [3]

$$WVTR = \frac{W_0 - W_1}{A \times 20} \left(\frac{g}{m^2 \times h}\right)$$

Where A is the area of permeation opening (m^2).

Results:

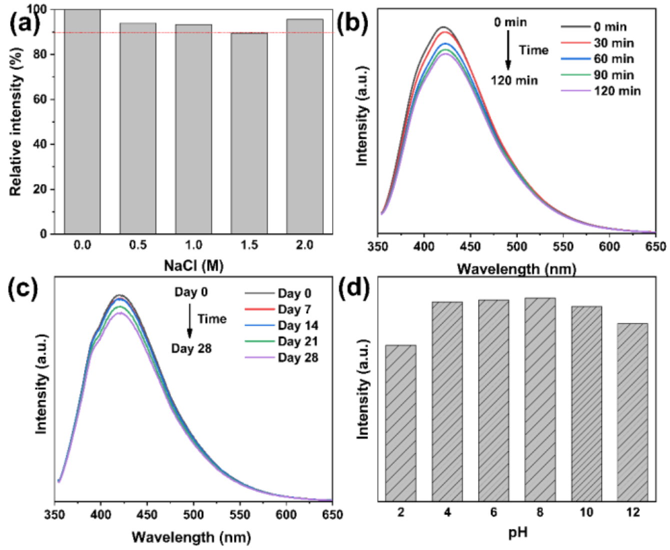

Figure S1. (a) Relative PL intensity (I/Io) of the synthesized CDs in NaCl solution with varying concentration (0.0–2.0 M), (b) PL spectra of the CDs under different irradiation times (0–120 min), (c) PL spectra of the CDs during storage, and (d) PL spectra of the CDs at different pH values (2.0–12)

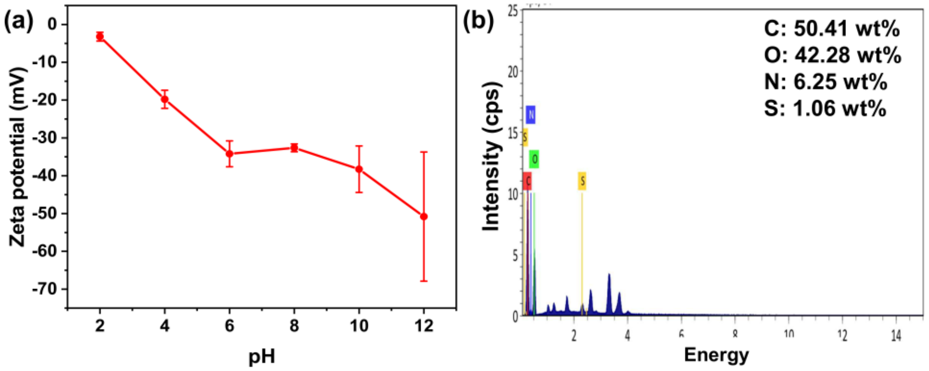

Figure S2. (a) Zeta potentials of CD solution at varying pH values, and (b) EDX spectrum of CDs. The EDX results were normalized to 100% after excluding trace amounts of ions (Ca, K, Cl, Si, Na, Mg).

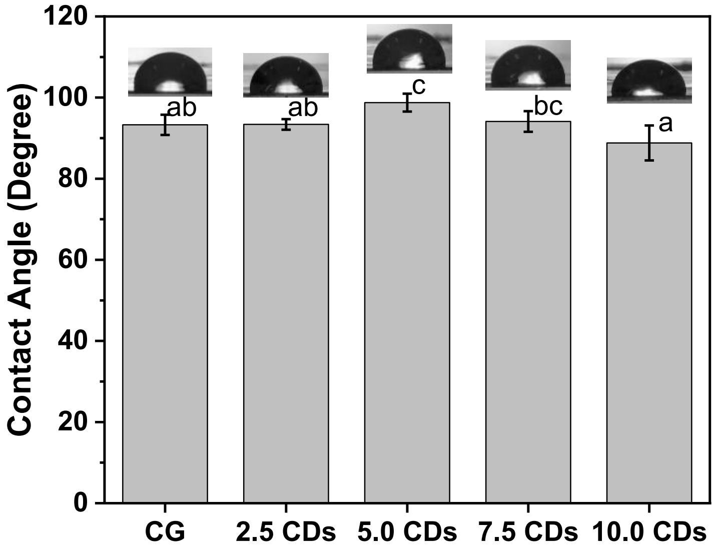

Figure S3. The contact angle of the chitosan/gelatin (CG) films incorporating varying amounts of the broccoli leaf-derived CDs (2.5–10%). The incorporation of CDs at 5% loading led to a slight improvement in the contact angle of the pristine CG film of ~6%

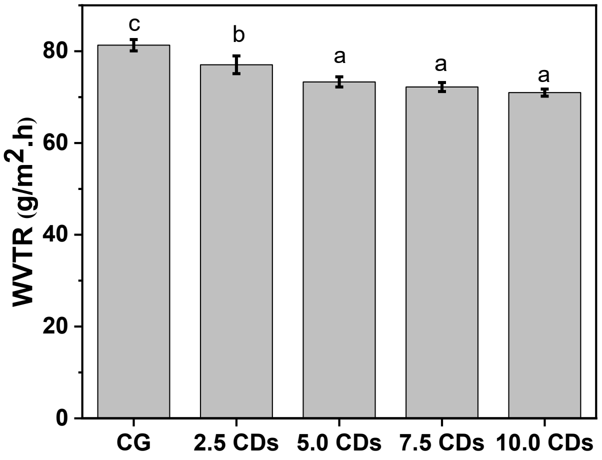

Figure S4. The water vapor transmission rate (WVTR) of the chitosan/gelatin (CG) films incorporating varying amounts of the broccoli leaf-derived CDs (2.5–10%). The incorporation of CDs led to a slight reduction in the WVTR of the pristine CG film of ~10%

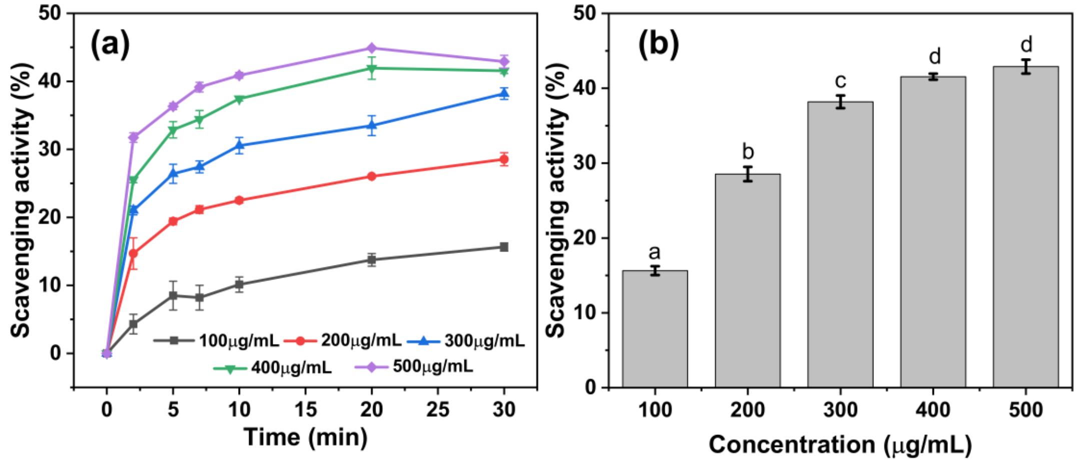

Figure S5. (a) DPPH scavenging activity of CDs at varying concentrations over time, and (b) DPPH scavenging activity of CDs at varying concentrations at 30 min. Different letters indicate statistical difference ($p < 0.05$)

References:

1. Lamour, G., et al., *Contact angle measurements using a simplified experimental setup.* Journal of chemical education, 2010. **87**(12): p. 1403-1407.
2. Manjua, A.C., et al., *Magnetic responsive PVA hydrogels for remote modulation of protein sorption.* ACS applied materials & interfaces, 2019. **11**(23): p. 21239-21249.
3. Sarwar, M.S., et al., *Preparation and characterization of PVA/nanocellulose/Ag nanocomposite films for antimicrobial food packaging.* Carbohydrate polymers, 2018. **184**: p. 453-464.
